# Evolutionary biology and genetic techniques for insect control

**DOI:** 10.1111/eva.12280

**Published:** 2015-07-15

**Authors:** Philip T. Leftwich, Michael Bolton, Tracey Chapman

**Affiliations:** ^1^ School of Biological SciencesUniversity of East AngliaNorwich Research ParkNorwichUK

**Keywords:** fitness, genetic modification, release of insects carrying a dominant lethal, resistance, selection, sterile insect technique

## Abstract

The requirement to develop new techniques for insect control that minimize negative environmental impacts has never been more pressing. Here we discuss population suppression and population replacement technologies. These include sterile insect technique, genetic elimination methods such as the release of insects carrying a dominant lethal (RIDL), and gene driving mechanisms offered by intracellular bacteria and homing endonucleases. We also review the potential of newer or underutilized methods such as reproductive interference, CRISPR technology, RNA interference (RNAi), and genetic underdominance. We focus on understanding principles and potential effectiveness from the perspective of evolutionary biology. This offers useful insights into mechanisms through which potential problems may be minimized, in much the same way that an understanding of how resistance evolves is key to slowing the spread of antibiotic and insecticide resistance. We conclude that there is much to gain from applying principles from the study of resistance in these other scenarios – specifically, the adoption of combinatorial approaches to minimize the spread of resistance evolution. We conclude by discussing the focused use of GM for insect pest control in the context of modern conservation planning under land‐sparing scenarios.

## Introduction

Insects spread disease and destroy millions of tons of crops each year. With global climate change and an ever‐increasing population size, there are significant challenges associated with safeguarding people from disease and maintaining food supplies. This provides an urgent stimulus to develop new methods for insect control. Traditional approaches include pesticides, integrated pest management, and biological control. However, each has serious drawbacks because of environmental and social costs and/or lack of (cost‐) effectiveness. For example, synthetic insecticides have been widely applied against a wide variety of pests and disease vectors – but their continual application selects strongly for resistance and is also nonselective, destroying natural enemies of the pests as well as perturbing the ecosystem as a whole. In addition, with increasing concerns of off‐target effects of pesticides, the range of chemicals available for control is diminishing.

In light of these concerns, and due to potential problems with existing methods, there has been increasing interest in applying genetic modification (GM) techniques for insect control (Thomas et al. 2000; Deredec et al. 2008). In these, the aim is to harness the natural mating system of the pest in order to introduce into the pest population traits that will ultimately lead to its demise. Genetic methods that are transmitted or inherited through one sex, and which sterilize, kill, or cause sex change in the other, offer the greatest control potential (Bax and Thresher 2009). A goal in developing and assessing new methods, and in refining existing ones, is to understand whether it is ultimately better to optimize control strategies over a range of different species, environmental, and biotic conditions, or instead to employ highly species‐ and/or environment‐specific targeting (see section Control strategies made evolution‐proof or evolution‐resistant, below; Leftwich et al. 2014).

Insect pests can be particularly difficult to control effectively using traditional nonselective methods such as biocides or insecticides if they are hard to target, or occur in close proximity with humans. For example, agricultural pests that exhibit flexible host use may have refugia across multiple host species, making them difficult to locate and eradicate. For pests in which the larvae reside within host fruits, the delivery of exogenously applied control agents may also be relatively ineffective at targeting the relevant life history stages. Insecticides used to treat disease vectors such as mosquitoes need to be accurately targeted because of the co‐occurence of the insect vector with human residences. However, mosquitoes such as *Aedes aegypti* are opportunistic in their breeding and resting sites; hence, finding all potential habitable spots for these insects may be difficult and labor intensive. Selective and species‐specific mechanisms, that is, those in which control is achieved when released males seek out wild females for matings, transmitting to them sterility or genes that kill offspring, therefore offer many advantages. A major one is that they rely upon the natural mating system of the pest, honed by natural selection over many millennia.

Broadly speaking, the control technologies that employ genetic mechanisms fall into two types: (i) those that act to suppress local populations and are themselves self‐limiting, eventually becoming extinct, and (ii) those in which the pest population is replaced by a more benign form or in which a trait is self‐sustaining and driven through the pest population to reduce the harm caused by the pest (Alphey 2014). These different strategies differ in the extent to which the introduced genes persist in the population. From an environmental perspective, the longer‐term impacts of these contrasting strategies upon the population structure and population genetics of the pest species involved may be very different, as discussed further below.

## Self‐limiting population suppression mechanisms

### Sterile insect technique

A key breakthrough in achieving effective and environmentally benign insect control was the introduction of the sterile insect technique (SIT) over half a century ago (Knipling 1955). This is a species‐specific method of insect suppression (Hendrichs et al. 1995; Krafsur 1998) in which insects are mass‐reared under factory conditions, sterilized by irradiation, and then released. The released males mate with wild females and these sterile matings lead to a reduction in the size of the pest population. SIT is generally more effective if only males are released (Rendon et al. 2004). This prevents the introduction into the population of females that can damage fruit crops or transmit disease and minimizes any reduction in the efficiency of suppression arising due to assortative mating among released individuals (rather than between released males and wild females). However, male‐only releases require that there is an efficient mechanism for sex sorting. SIT males must be able to seek out females and mate. However, SIT males are typically less competitive than wild males because of their history of adaptation to factory, rather than natural, conditions and because the irradiation used for sterilization significantly reduces fitness (Briceño and Eberhard 1998; Briceño et al. 2002; Lux et al. 2002; Parker and Mehta 2007). This reduced competitiveness can be mitigated to some extent by releasing more insects and increasing the overflooding ratio. Therefore, SIT males are often released periodically in large numbers to flood the resident population in order to achieve control. SIT has had great successes – but also some failures and trials in which only limited success was reported (e.g., in some mosquito trails conducted in the 1970s, reviewed by Benedict and Robinson 2003).

SIT has been used with success against the New World screwworm fly (Krafsur 1998), melon fly (Iwahashi et al. 1983; Kuba et al. 1996; Koyama et al. 2004), medfly, and tsetse fly (Hendrichs et al. 1995). There are, however, acknowledged problems, which may be responsible for examples in which SIT has not been successful or has had limited impact (e.g., Benedict and Robinson 2003). Sterilization by irradiation is perhaps the most significant problem because of the deleterious impact it has on the fitness of the released insects. A lower irradiation dose can be used to reduce harmful effects on fitness, but will allow some fertile individuals to be released. The sterilization dose used therefore needs to balance the degree of sterilization achieved versus its fitness impact. Additional problems are the deleterious side effects caused by ‘domestication’ of wild strains during mass rearing, leading to poor field performance of released males (Cayol 2000).

As noted above, ‘male‐only’ releases are advantageous for control (Rendon et al. 2000; Rendon et al. 2004), provided that efficient sex‐sorting mechanisms can be achieved, because they reduce the collateral damage caused by the release of sterile females (e.g., fruit ‘stings’ or biting) and prevent matings among released insects that have zero control value. Efficient sex‐sorting mechanisms can reduce the impact of some potential drawbacks of SIT, such as the evolution of ‘behavioral resistance’, that is, discrimination by wild females against mating with SIT males (e.g., McInnis et al. 1996) and the evolution of changes in the timing of mating that lessen the probability of matings between released and wild flies (e.g., Economopolous et al. 1971; Economopolous 1972; Miyatake and Shimizu 1999). However, poor reliability of sex‐sorting mechanisms for such male‐only releases can result in additional problems for maintaining productivity and release strain stability (Seawright et al. 1978; Papadopoulos et al. 1998; Hendrichs et al. 2002; Lux et al. 2002; Robinson et al. 2002; Barry et al. 2003; Mossinson and Yuval 2003; Robinson et al. 2004; Windbichler et al. 2008). An increased frequency of remating by wild females mated to sterile SIT males, which can significantly reduce the effectiveness of SIT, is also possible (Kraaijeveld and Chapman 2004). Together these factors can explain examples of poor field performance and/or mating discrimination against SIT males (McInnis et al. 1996; Cayol 2000).

### Release of insects carrying a dominant lethal

To circumvent problems identified with the application of SIT, there has been intense interest in GM technologies (Handler and James 2000; Heinrich and Scott 2000; Thomas et al. 2000; Horn and Wimmer 2003; Robinson et al. 2004; Fu et al. 2007). One such method that has been developed for a range of different pests and tested in laboratory through to open field conditions is the ‘release of insects carrying a dominant lethal’ (RIDL; Thomas et al. 2000). RIDL offers potentially significant improvements over SIT (Schliekelman and Gould 2000; Thomas et al. 2000) perhaps most importantly because it circumvents the need for sterilization using irradiation. All fitness costs of irradiation are therefore eliminated. RIDL technology can target both sexes, but as noted above, the delivery of sex‐specific action offers significant benefits for control. As an example of the application of RIDL technology, the female‐specific (fs) ‘fsRIDL’ system (Fu et al. 2007) induces female‐specific lethality through alternative splicing of sex‐specific introns, leading to the production of a tetracycline‐repressible transactivator fusion protein (tTA) in females, resulting in a lethal tTA positive feedback loop. Adding tetracycline to the diet suppresses lethality – but in the wild, the lethality is expressed and kills females at the pre‐adult stage.

Transgenic RIDL insects have now been produced for pests of economic (Gong et al. 2005; Ant et al. 2012; Jin et al. 2013) and medical (e.g., Phuc et al. 2007; Fu et al. 2010; Harris et al. 2011) importance. Using the female‐sterile system, male‐only releases are easily achieved by removing tetracycline from the diet in the release generation. fsRIDL therefore offers a simple way to reduce or eliminate females in the pest population. In addition, the use of autofluorescent markers for transformation (Horn et al. 2002; Fu et al. 2007) facilitates the detection of released individuals in the field. Caged and field‐caged trials of RIDL medfly (*Ceratitis capitata*; Leftwich et al. 2014), olive fly (*Bactrocera oleae* Gmelin; Ant et al. 2012; Harvey‐Samuel et al. 2014), diamondback moth (*Plutella xylostella* L.; Jin et al. 2013), mosquitoes (*Aedes aegypti*; Phuc et al. 2007; Wise de Valdez et al. 2011), and pink bollworm (*Pectinophora gossypiella*; Jin et al. 2013) show the potential for RIDL strains to eliminate or control the spread of wild‐type populations. More recent tests of RIDL strains are now employing more complex setups and following fitness outcomes in multigenerational designs (e.g., Harvey‐Samuel et al. 2014). These studies have the potential to highlight sensitivities of strains that are not apparent under simpler glass house or laboratory tests. This approach could be further expanded in the future to capture likely performance under an ever‐broader range of ecological and environmental conditions. Despite the findings that GM strains may sometimes show evidence of reduced competitiveness in direct comparisons with wild types, there are nevertheless many examples of their potential effectiveness to control pest populations (Thomas et al. 2000; Fu et al. 2007; Harris et al. 2011; Wise de Valdez et al. 2011; Ant et al. 2012; Jin et al. 2013; Harvey‐Samuel et al. 2014; Leftwich et al. 2014). Any fitness loss of the GM strains can normally be countered by procedures such as increasing the frequency or number of released individuals.

RIDL technology is advanced in terms of its application under open field conditions in comparison with other GM control strategies. For example, strains of RIDL mosquitoes (Phuc et al. 2007) have already been subject to open field testing in the Cayman Islands (Harris et al. 2011, 2012), Malaysia (Lacroix et al. 2012), and Brazil (Alphey 2014). In these field trials, the released male insects were found to persist in the environment, to locate wild females and successfully mate with them and to achieve pest population suppression (Harris et al. 2011, 2012; Alphey 2014). Genetically sterile RIDL *A. aegypti* strains have also been tested under field release conditions. They show similar field longevity and maximum dispersal distances to a progenitor strain, but exhibit reduced mean dispersal distances (Lacroix et al. 2012). The potentially reduced flight potential of RIDL insects such as mosquitoes should be considered when developing facets of the release programs such as release sites and release densities (Bargielowski et al. 2011).

The success of GM technology itself depends on the effectiveness of the construct in killing, whether its effects are sex‐specific (e.g., Fu et al. 2007), the life history stage at which it kills (e.g., Phuc et al. 2007) the stability of the transgene construct, the stability of the insertion, any fitness costs arising from insertion of the construct, and any fitness costs of the expression of the construct. The killing potential of the strains for potential release can be isolated in initial laboratory testing, as can the exact life history stages affected (Thomas et al. 2000; Fu et al. 2007). The life cycle stage that is targeted depends upon the specific pest and the reagents available. For example, for RIDL programs against agricultural pests in which larvae live within commercially important crops, early‐acting lethality might be advantageous to limit larval penetrance into fruits and hence reduce spoilage. However, under female‐sterile programs (e.g., Fu et al. 2007), any such benefit is negated as male RIDL larvae survive and continue to damage fruit (Leftwich et al. 2014). In contrast, for non‐RIDL programs that target vectors of disease such as mosquitoes, transgenes that act to reduce the probability of disease transmission should ideally be much later acting in order to enhance additional control arising from increased density‐dependent mortality among larvae (which do not themselves cause disease, e.g., Wise de Valdez et al. 2011).

Stability of GM construct locations in the genome can be achieved by removing the mechanisms or sequences needed for the gene carriers (e.g., transposable elements) to remobilize (Dafa'alla et al. 2006). Internal stability of the GM constructs themselves is also important to avoid breakdown of the mechanisms they deploy. The potential for such breakdown can be assessed using stress tests of GM strains, subjecting them to heat and food stresses and testing whether killing ability is compromised. Fitness costs arising from the insertion site of constructs causing mutagenic effects in the host genome are normally circumvented by producing and comparing multiple lines with different insertion sites and then selecting those with the least impact on performance. Docking mechanisms to introduce constructs (e.g., Nimmo et al. 2006) into the same, low fitness impact, genomic location each time (similar to the ‘Gateway’ technology) would be a useful development for the future.

Fitness effects associated with the expression of the transgenes (e.g., of markers) that are separate from the killing effects are also possible. These can be measured under controlled conditions by comparing the fitness of individuals bearing the transgenes in the activated or nonactivated form. Although it is possible to do this in practice, it has proved more fruitful to compare the overall performance and fitness of the GM in comparison with progenitor (e.g., Massonnet‐Bruneel et al. 2013) and/or wild‐type strains (e.g., Leftwich et al. 2014). These tests combine the sum of the effects noted above as well as any deleterious effects arising from the process of domestication (Table**** 1).

**Table 1:** Selection on focal traits arising from rearing under laboratory or factory conditions and potential strategies to minimize deleterious impacts for insect control

Focal trait(s)	Direction and nature of selection applied in the laboratory or factory	Strategies to minimize deleterious impact on control potential
*Traits under selection in laboratory or factory*		
Diet utilization	Strong selection for high productivity on novel artificial diets, likely to select for adaptation to utilize new food components efficiently and to affect many different life history traits (e.g., Hood‐Nowotny et al. 2012; Yahouedo et al. 2014). Adaptation to artificial, standardized, and simpler diets is expected to reduce gut microbial diversity (Behar et al. 2008a; Chandler et al. 2011), reducing host fitness. Dietary changes could also alter pheromone components, thus affecting reproductive success (e.g., Sharon et al. 2010). Diet can also deleteriously impact on gut microbial diversity if antibiotics are added (Behar et al. 2008a,b; Ben Ami et al. 2010), for example, to diets in laboratory or factory settings to suppress dominant lethality (e.g., Thomas et al. 2000)	Use of more complex natural and varied diets or diet supplements (e.g., Kaspi and Yuval 2000). Use of probiotics to restore gut microbial diversity (e.g., Niyazi et al. 2004; Gavriel et al., 2011)
Inbreeding	Genetic bottlenecks that occur upon adaptation of the pest species to the mass‐rearing conditions may reduce genetic diversity (e.g., Cayol 2000; Ciosi et al. 2014; Parreno et al. 2014)	Can be countered by periodic introduction of ‘fresh blood’ into mass‐rearing strains and therefore releasing individuals with greater genetic diversity (e.g., Cayol 2000; Gilchrist and Meats 2012)
Development time	Likely to be selection for rapid development, but depends upon timing of pupal collections for seeding the next parental generations. A genetic correlation between development time and mating traits is reported. Hence, selection on development time can lead to correlated selection for altered timing of mating, with the potential to result in reproductive isolation between wild and factory strains (e.g., Miyatake and Shimizu 1999)	Avoid collection and use of only the first pupae to emerge to propagate the next generation
Larval density	Selection for success under elevated larval density. Variation in larval density has the potential to affect body size (e.g., Medici et al. 2011) and survival (e.g., Marti and Carpenter 2008; Medici et al. 2011) and hence has multiple effects on fitness	Could reduce larval densities during culturing to a level with minimal impact on body size. However, given that reduced density may also increase costs and reduce overall efficiency, one could instead optimize density and size across potential trade‐offs between overall effectiveness/efficiency/cost (although this optimum is harder to measure)
Time to sexual maturity	Selection for rapid sexual maturity and first egg laying (e.g., Miyatake 1998; Hernandez et al. 2014), because those individuals that mature quickly contribute more to the next generation	Avoid taking the very first fertilized eggs that are laid. Use of other measures such as avoidance of selection for rapid development and small body size
Body size	Selection on body size is possible depending upon diet and development time regimes chosen (e.g., Cayol 2000; Cendra et al. 2014). Avoidance of inadvertent selection for small body size likely to be important and large male body size generally associated with increased mating success (e.g., Rodriguero et al. 2002). Larger females may also be more fecund. Changes in body size may also alter blood‐feeding rates in disease vectors such as mosquitoes, with smaller females feeding more often (e.g., Nasci 1986; De Xue et al. 1995; Farjana and Tuno 2013) . Variation in body size could therefore potentially alter the probability of disease transmission	Monitor body size, adjust diet, and development time regimes if practical (e.g., Cayol 2000)
Longevity/life expectancy	Longevity *per se* is not expected to be a target of direct selection under mass rearing, but is likely to change as a side effect of changes to development time, body size, oviposition behavior and timing (e.g., Cayol 2000; Hernandez et al. 2014)	Changes to longevity and life expectancy will be minimized by measures to reduce selection for divergent traits under mass rearing (Cayol 2000)
Oviposition	Strong selection for a different type of oviposition behavior in comparison with the field, into artificial diets or through artificial egg laying devices. Likely to alter oviposition behavior substantially and select for traits such as an earlier, shorter, and more productive oviposition period (e.g., Suenaga et al. 2000; Hernandez et al. 2014)	Use of natural host‐mimicking devices for egg laying in addition to artificial ones, although operational constraints may render such enrichment impractical
Productivity	Selection for high fecundity (e.g., Hernandez et al. 2014) and productivity arising from requirement for sufficient numbers of pupae to release	May be difficult to address by itself, although implementation of all the other measures could help minimize this problem
Courtship behavior	Crowded conditions and adaptation to mass rearing are likely to select for truncated courtships (e.g., Briceño and Eberhard 1998; Briceño et al. 2002), alterations to courtship songs (e.g., Briceño et al. 2009), increased courtship interruptions (e.g., Briceño et al. 2002; Briceño and Eberhard 2002), more male–male mounting (e.g., Gaskin et al. 2002; Weldon 2005), and potentially altered courtship thresholds in females (e.g., Briceño et al. 2002)	Reduce density of adult cages and increase complexity of the environment, to the extent practical. Could consider reducing the number of adult males recruited to the cages (Leftwich et al. 2012) to reduce intensity of male–male competition
Pheromones	The use of artificial diets and mass‐rearing conditions may be associated with alterations to pheromones (e.g., Sharon et al. 2010; Benelli et al. 2014). The close proximity of females may also select for differences in male pheromone strategies. Large numbers of pheromone‐fanning males within large cages are likely to result in a pheromone ‘fog’. This may lead to selection for decreased pheromone emission. Individuals may also become desensitized to pheromones (Briceño et al. 2002; but see Kuriwada et al. 2014)	Reducing densities within adult cages to the extent that is practical. Consider periodic selection for ability to produce (males) and track (females) pheromones (e.g., wind tunnels). Diet enrichment to promote production of diverse pheromone blends (e.g., Kaspi and Yuval 2000; Niyazi et al. 2004; Gavriel et al., 2011)
Male–male competition and female mate choice	Crowded conditions and adaptation to mass rearing are likely to select for intense male–male competition leading to divergent mating strategies in comparison with the wild type. Frequent disturbance and potentially truncated or reduced thresholds for female choice decisions are also expected (see courtship behavior, above)	Reduce densities within adult cages and increase complexity of the environment (e.g., Liedo et al. 2007) to the extent practical
Mating frequency	Crowded conditions likely to select for more frequent matings and rematings (e.g., Vera et al. 2003; Kraaijeveld et al. 2005). This may select for changes in male ejaculate allocation and competition strategies (e.g., Linklater et al. 2007) and for other mating behaviors that are distinct from those that occur in the field	Reduce densities within adult cages and increase complexity of the environment (e.g., Liedo et al. 2007) to the extent practical
Assortative mating	Not evident under domestication, there is the potential for assortative mating to occur if there are changes to the sexually selected traits listed above. This could result in resistance of released males to mate with wild females (e.g., McInnis et al. 1996). Assortative mating due to evolved differences in the time of mating is also possible (Economopolous et al. 1971; Economopolous 1972; Miyatake and Shimizu 1999). A different kind of problem may be ‘mating failure’ between released males and wild females (e.g., Perez‐Staples et al. 2013)	Assortative mating (and damage arising from female release) can be eliminated through the use of single‐sex release programs (e.g., Hendrichs et al. 1995), if practical and cost‐effective. Avoid selection for traits that increase reproductive success under laboratory‐/factory‐specific conditions. Increase overflooding ratios of released males into the wild. Increase diversity of age classes of male introduced into the wild (e.g., Gilchrist and Meats 2012)
Living in a simpler environment	Laboratory and factory conditions are simple environments that lack many of the important complexities of field environments (even ‘simpler’ ones such as agricultural environments)	Behavioral enrichment, for example, artificial lekking/perching sites, more horizontal surface area (e.g., Liedo et al. 2007). Artificial trees/host plants
*Traits not under selection in laboratory or factory conditions*		
	Selection for flight ability is minimized	Use of large, lower density cages, consider periodic selection for flight ability (e.g., use of flight tunnels)
	Selection for long‐range mate finding is minimized	Use of large, lower density cages, consider periodic selection for mate finding ability (e.g., use of flight tunnels with pheromone release). Use of parapheromones and other chemical agents (e.g., Shelly 1995; McInnis et al. 2011; Benelli et al. 2014) to enhance male mating success
	Selection for predator evasion	Hard to achieve, but general increases to competitiveness of released individuals might increase agility and hence predator evasion
	Selection for disease resistance and avoidance of trade‐offs diverting resources from mate finding to combating infection if disease is encountered by individuals released into the field	Hard to achieve other than by periodic reintroduction of wild‐type genetic variation

### Probiotics to enhance SIT and RIDL performance

It is now widely recognized that a significant contribution to host fitness comes from associations with commensal gut bacteria (the gut microbiome; Dillon and Dillon 2004). As in vertebrates (Turnbaugh et al. 2006; Vijay‐Kumar et al. 2010), the gut microbiome in invertebrates can have widespread and significant effects on fitness (Dillon and Dillon 2004; Ben‐Yosef et al. 2008a). In pest and nonpest fruit flies, changes in the gut microbiome can alter life span, mate choice, reproductive physiology, development, and metabolism (Behar et al. 2008a,b; Ben‐Yosef et al. 2008a,b; Sharon et al. 2010; Shin et al. 2011). The number and diversity of gut bacteria of laboratory‐ and mass‐reared pest and nonpest fruit flies is diminished in comparison with wild flies (Ben Ami et al. 2010; Chandler et al. 2011). Hence, there is evidence that the gut microbiome changes significantly during domestication. While the diet can alter the composition of the gut microbiome to some extent, there is an emerging picture that there are core members of this community irrespective of diet. Almost nothing is currently known, however, about how these core components colonize the gut, the role of the host in that process and transmission routes. With these factors in mind, attention has turned to the potential for probiotic treatments to improve sterile SIT male reproductive performance (Gavriel et al. 2011).

Changes to the gut microbiome are of particular interest for GM strains such as those using the RIDL technology, which all utilize tetracycline‐repressible promoters (e.g., Thomas et al. 2000; Alphey 2002; Alphey and Andreasen 2002). As noted above, in these strains, the lethality or manipulated gene expression is under the control of a tetracycline‐repressible promoter. The effects of the construct are suppressed during normal culture in the laboratory or factory using dietary tetracycline (e.g., Fu et al. 2007; Phuc et al. 2007). The continual exposure of RIDL strains to antibiotics is likely to (i) alter the composition of gut bacterial communities through a reduction in gut bacterial diversity and (ii) select for tetracycline‐resistant gut bacteria. The effect on host fitness of gut bacterial communities that are altered in these ways is not yet known.

It is therefore important to understand whether any loss of gut bacteria in domesticated laboratory strains and in those maintained on antibiotic diets can be slowed or reversed by variation in dietary regimes or supplementation with bacteria in the diet. That such ‘probiotic’ treatments have significant promise is shown by experiments in which the reproductive performance of sterilized male medflies was improved by diet supplementation with *Klebsiella oxytoca* (Gavriel et al. 2011).

### Incompatible sterile matings

The sterile‐male incompatible insect technique (IIT) can lead to a type of population suppression that is similar, in principle, to SIT and RIDL (Brelsfoard and Dobson, 2009; Laven 1967). It can be conferred by incompatible matings between individuals infected/not infected by strains of maternally inherited intracellular bacterial parasites such as *Wolbachia* (Brelsfoard and Dobson, 2009). Control is achieved through the cytoplasmic incompatibility phenotype (CI) that occurs when *Wolbachia*‐infected males mate with uninfected females resulting in female sterility. The exact mechanism is still not fully known, although it results in early development arrest in the embryos produced from incompatible matings.

Control could therefore be achieved if *Wolbachia*‐infected males were released into a non‐*Wolbachia*‐infected population to mate with noninfected females. This strategy has been considered for several insects, including mosquitoes and medflies (Brelsfoard and Dobson, 2009; Zabalou et al. 2009). It has been realized that, in mosquitoes, males can be released without increasing the number of biting insects (only females bite and transmit disease), and because *Wolbachia* is inherited solely through the maternal line, the released insects do not spread *Wolbachia* through the population, and hence, males represent an evolutionary ‘dead end’ (O'Connor et al. 2012). It is important under this control scenario that no infected females are released, as all matings with infected females are compatible (indeed, this is the mechanism for driving *Wolbachia* through populations by gene driving, see below). The risk of simply contributing to the expansion of the pest population can be reduced if the target population is also infected but with a different strain of *Wolbachia*, giving bidirectional CI and sterility in eggs resulting from both types of matings that could occur.

The potential success of this strategy was first demonstrated in *Culex quinquefasciatus* mosquitoes many decades ago (Laven 1967). However, it was thought not to be generally applicable because there were perceived to be limited numbers of examples of bidirectional CI. However, with increased ability to artificially transfect species (e.g., with *Wolbachia* strains), this technique may now offer new opportunities for control. For example, this type of self‐limiting control using *Wolbachia* has been observed in *Aedes polynesiensis* mosquitoes. This species carries a natural, single‐strain *Wolbachia* infection. Release of males artificially transfected with a different *Wolbachia* strain derived from another *Aedes* species resulted in successful bidirectional incompatibility with the wild‐type *Aedes polynesiensis* population, including in open field tests (Brelsfoard et al. 2008; O'Connor et al. 2012).

## Population replacement or introduction of traits that reduce the deleterious impact of the pest

For population replacement to confer insect control, mechanisms to drive genes through populations to effect control are needed. Driving mechanisms are required in which genes exhibit non‐Mendelian transmission, to enhance their own representation above that of other genes in the genome. Several such driving genes or systems are known, including *Wolbachia*‐based (Hoffman et al. 2011), homing endonuclease genes (HEGs; Burt 2003), and transposable element‐based systems (e.g., *Medea*). We focus in this section primarily on the *Wolbachia* and HEG systems. The recently described ‘mutagenic chain reaction’ (MCR) system conferred by CRISPR gene editing is discussed in the following section on newer technologies.

Key to successful invasion of traits that will lead to control of the target pest is an understanding of the ease of driving genes conferring the control trait through the population. The initial establishment and spread of drive is the crucial step and depends on many factors which sum to a property known as the ‘invasion threshold’. However, some drive systems can theoretically spread from any initial frequency (Deredec et al. 2008; Alphey and Bonsall 2014) although stochastic effects are expected to be significant at low initial frequencies (which will be true for any type of release program). Whether the invasion threshold, if it exists, is high or low determines the size and frequency of the initial inoculum into the pest population required to achieve control (Alphey 2014). These issues are not unique to drive‐based systems, and overflooding thresholds for achieving suppression are also critical for success in the SIT and RIDL methods described above. These ratios determine whether the released flies reduce damage to below the relevant economic threshold or disrupt disease transmission efficiently.

### Driving refractoriness to pathogen transmission using *Wolbachia*

One of the best‐known gene drive systems is that, associated with *Wolbachia*, a maternally inherited intracellular parasite. *Wolbachia* infection can result in a number of different driving phenotypes such as male killing and cytoplasmic incompatibility (CI), depending on the species infected and *Wolbachia* strains involved. It is the CI phenotype that offers the potential for control because, through females, it can drive *Wolbachia* infection (and any control potential offered by the parasite) through populations. *Wolbachia*‐infected females have a substantial fitness advantage over uninfected females (which become sterile following matings with infected males) and given that the *Wolbachia* parasite is maternally inherited, and this will result in an increase in *Wolbachia* in the population as a whole (Turelli and Hoffmann 1995).

To date, *Wolbachia* infection has been used to control disease (e.g., dengue virus) transmission in mosquitoes. *Wolbachia* infection is known in mosquitoes to interfere (by as yet unknown mechanisms) with the efficiency with which hosts can transmit pathogens such as dengue virus. Hence, the driving of *Wolbachia* through such species using CI can potentially reduce the spread of disease (Hoffman et al. 2011; Yeap et al. 2011). A proof of principle for insect control by this method comes from the spread of a strain of *Wolbachia* derived from *Drosophila* through natural populations of *Aedes aegypti* in Australia (Hoffman et al. 2011). Similarly, *Wolbachia*‐induced refractoriness to the spread of *Plasmodium* by the mosquito *Anopheles stephensi* has also reported (Bian et al. 2013). More recently, improvements to the potential spread and penetration of *Wobachia* into natural populations are proposed by linking the introduction of *Wolbachia* to insecticide resistance (e.g., Hoffmann and Turelli 2013).

### Homing endonucleases

Homing endonuclease genes (HEGs) are found naturally among fungal genomes and represent a potentially powerful mechanism for driving genes through populations to achieve insect control (Burt 2003; Deredec et al. 2008). Although the primary focus is on HEG as gene drivers, it should be noted that self‐limiting forms of HEG control are also possible (Burt 2003). In the heterozygous state, the protein encoded by HEG genes causes a double‐stranded break to occur in the homologous chromosome at the same position. If the break is repaired using the HEG‐bearing chromosome as template, the HEG becomes homozygous as a result of gene conversion or homologous recombination. This mechanism therefore represents a powerful means for driving genes through populations, using HEGs as vehicles. In agricultural pests, potential control agents that could be loaded into HEGs are genes that decrease viability or that decrease female fecundity or distort the sex ratio. The latter could be especially effective, for example, if HEG activity could be restricted to the male germ line but act on female‐specific traits or inactivate or degrade the X chromosome.

Proof of principle experiments for insect control via engineered HEGs has been conducted in the fruit fly *Drosophila melanogaster*, in which sperm development and the female germ line were targeted by the HEG I‐*SceI* (Chan et al. 2011). HEG‐derived drive has also been shown *in vivo* using the same drive gene in *Anopheles gambiae* mosquitoes, where it appears to occur at much higher efficiency (Windbichler et al. 2011). This is partly because in *D. melanogaster*, the homologous recombination needed for the drive to occur appears to be restricted to specific sperm cell stages within the testis. However, the efficiency of HEG drive can, in principle, be improved by trialling different genetic constructs. The overall efficiency of HEG drive is also significantly affected by temperature (Chan et al. 2013), which will be an important consideration if this technology moves into field trials.

The effects of population genetics upon the spread of HEG‐based systems have also been investigated using theoretical approaches (Alphey and Bonsall 2014). The results show that the success of HEG‐based drive depends critically upon the interaction of population genetic and ecological factors such as density‐dependent effects during larval competition, the timing of the impact upon fitness of HEG drive, and the relative fitness of the different wild‐type and HEG genotypes present in the population.

## Control potential of new, or underutilized, techniques

### CRISPR and the mutagenic chain reaction

A new, and potentially revolutionary, gene drive system recently gained attention in the context of insect control (Esvelt et al. 2014), with a recent study in *D. melanogaster* reporting 97% transmission (i.e., well over the expected 25% Mendelian outcome) of a normally recessive, loss of function *yellow* pigmentation gene (Gantz and Bier 2015). This was achieved using the increasingly popular CRISPR gene‐editing tool (Jinek et al. 2012) to create a ‘mutagenic chain reaction’ (MCR). The transmission efficiency reported by this new method far exceeds that which can currently be achieved with the HEG strategies described above and this technology therefore offers a highly potent prospect for gene drive control. The MCR technique used was, however, criticized on the basis of its lack of safeguards (Bohannon 2015). The editing and targeting sequences were contained within the same gene cassette, meaning that there was no way to stop or ‘recall’ the gene drive once initiated. However, such safeguards can be built in and, with such efficient driving, the possibility to drive through subsequent neutralizing genes should also be considered.

### Control through gene manipulation via RNA interference

Concerns about the use of GM technologies, and variation in the length of time needed to address regulatory concerns in different countries, have prompted interest in the use of RNA silencing to produce sterile males for control releases (e.g., Thailavil et al. 2011; Whyard et al. 2015). Such methods are currently considered non‐GM technologies. The RNA silencing method relies on the introduction into the target insects of double‐stranded RNA that is complementary to the endogenous gene to be silenced. The double‐stranded RNA (dsRNA) then catalyzes the degradation of the target RNA via the RNA interference (RNAi) mechanism (reviewed by Bartel 2004). dsRNA can be introduced into invertebrates via feeding or injection and exert a significant silencing phenotype. There are several possibilities for control, including the silencing of testis‐expressed genes in order to sterilize males or to manipulate genes in the sex determination pathway in females, for example, to change females into sterile pseudomales (e.g., Thailavil et al. 2011; Whyard et al. 2015). A recent study fed dsRNA to larvae of *Aedes aegypti* mosquitoes and showed reduced fertility in groups in which male testis genes were silenced and an increase in the number of males: females in groups in which female‐specific *doublesex* RNA was targeted (Whyard et al. 2015). Key to success of RNAi for control will again be the relative competitiveness of the released insects, the efficiency of sterilization (to minimize the release of fertile males), cost, and the likelihood of resistance evolution.

### Insect control through reproductive interference and the actions of seminal fluid proteins

Incomplete mate recognition, leading to reproductive interference in matings between closely related species, is of core interest in evolution and ecology because of its role in maintaining species barriers. It may often also be asymmetric (when reciprocal interspecific matings incur different fitness costs). This ‘satyrization’ has long been considered of potential interest in insect control because of its potential to result in competitive displacement of species (DeBach 1966). For example, there is the potential for control if an insect vector exhibiting low disease transmission characteristics could be introduced to replace a resident species with high disease causing potential.

Such a phenomenon is thought to have occurred in the USA in mosquitoes of the genus *Aedes*. *Aedes aegypti*, a major vector of dengue virus, suffered competitive exclusion following the spread over the last 3 decades of the Asian tiger mosquito *A. albopictus* (Bargielowski and Lounibos 2014). *Aedes albopictus* itself can carry and transmit dengue and chikungunya viruses, although it is generally thought to represent a lower risk to human health. Hybrid matings are costlier to *A. aegypti* than to *A. albopictus* females, as seminal fluid proteins (Sfps) from *A. albopictus* males transferred into *A. aegypti* females render the latter refractory to conspecific matings (Tripet et al. 2011). There is no such effect in the reciprocal mating, conferring the observed asymmetry in fitness costs. This asymmetry and the associated costs of hybrid matings predict selection for rapid evolution of reproductive character displacement in areas where the two species occur in sympatry, to prevent such matings. Interestingly, evidence for just this phenomenon has recently been described (Bargielowski et al. 2013). Asymmetry in fitness following hybrid matings across many species of *Drosophila* is well known (Coyne and Orr 1989). However, the contribution of Sfps in this context has not been studied, even though it was first described decades ago (Fuyama 1983). Further research into the potential for control via reproductive interference could therefore be useful. A potential problem for insect control under satyrization, however, is that successful competitive exclusion could select for resistance, leading to the potential reinvasion of the pest.

The biodiversity and potential control toolkit represented by Sfps is extensive. These molecules vary hugely in structure (Mueller et al. 2005) and function (Ram and Wolfner 2007) and cause a profound remodeling of female behavior and physiology following their transfer during mating (e.g., Chapman 2001; Sirot et al. 2014). They can alter female sexual receptivity, ovulation and egg laying, feeding and sleeping, sperm storage, retention and usage, and immunity gene expression (Sirot et al. 2014). These phenotypes have significant effects on fitness (Chapman et al. 2003; Chapman 2006) and some genes that encode Sfps evolve extremely rapidly (Swanson et al. 2001; Clark and Swanson 2005).

In pest species such as medflies Sfp transfer can alter female behavior from that associated with seeking mates to that associated with searching for oviposition sites (Jang et al. 1998). This offers the potential for self‐limiting control strategies in which females might be prevented from switching on behaviors associated with crop damage (egg laying). There has also been much research on Sfps in *Anopheles* mosquitoes (e.g., Baldini et al. 2013; Gabrieli et al. 2014; Shaw et al. 2014). These studies offer much in the way of raw material for exploring new control strategies (Davies and Chapman 2006). The potential for Sfp engineering, perhaps combined with asymmetric reproductive interference, is so far relatively untapped and could offer useful complementary additions to the control strategies described above. It is worth anticipating that, as with other methods, those based upon Sfp engineering have the potential to become compromised by the evolution of resistance (e.g., behavioral resistance against mating with Sfp‐manipulated males). Strategies to mitigate such effects should therefore be simultaneously considered.

### Underdominance for driving control mechanisms

Underdominance occurs when the fitness of a heterozygote is lower than for both corresponding homozygotes. In theory, this can be used to drive an underdominant transgenic construct into a population to replace wild‐type alleles (Davis et al. 2001; Altrock et al. 2010; Reeves et al. 2014). The likelihood of population allele replacement depends upon the initial frequency of introduction and does not require that both wild‐type and introduced homozygotes have equal fitness, just that both their fitnesses are greater than the heterozygote. Such a system would be geographically limited and reversible (by reintroduction of the wild‐type allele), hence represent a self‐limiting form of control.

The principle of insect control through drive resulting from underdominance has been around for decades. However, a recent study successfully developed proof of principle in *D. melanogaster* (Reeves et al. 2014). The expression of a *Minute* locus was knocked down in heterozygotes. In this, RNAi was used to knock down the expression of one of the many *Minute* loci. *Minutes* are haplo‐insufficient; therefore, the knockdown resulted in a dominant, deleterious fitness effect (significantly delayed development, small size). The transgenic homozygote was rescued from this effect by the inclusion of a rescue gene to elevate the level of the Minute transcript to a functionally wild‐type level. The introduction of the underdominant transgene caused successful replacement of the wild‐type allele in as little as 5 generations in laboratory population tests. The introduction of transgenes that render hybrid matings costly could though select for the rapid evolution of mating barriers between the wild type and transgenics, which might reduce its efficiency.

With the ever‐increasing opportunity to design constructs for greater stability and efficiency, further work into these new or under‐employed genetic mechanisms might be very useful in light of the findings that they have at least the potential for efficient gene drive.

## Risks of existing and new technologies

The risks of the various control methods and mitigation strategies are discussed elsewhere (e.g., Alphey 2014; see also Bohannon 2015) and summarized only briefly here. The relative risks are generally held to be lower for suppression in comparison with replacement or driving mechanisms. This is because suppression mechanisms are inherently self‐limiting and drive themselves extinct, whereas driving mechanisms have greater persistence and longer‐term consequences should the technology fail. RIDL technology is further advanced than any of the other current GM control methods and has been successfully subjected to laboratory greenhouse, field cage, and open field trials (Wise de Valdez et al. 2011; Ant et al. 2012; Harris et al. 2012; Jin et al. 2013; Harvey‐Samuel et al. 2014; Leftwich et al. 2014). The open field release of GM insects is not without controversy, and any such release obviously requires extensive licencing, technical, regulatory, and public engagement activity to investigate the safety of the technology in terms of to the environment and human health. Public engagement activities are also essential to inform and address potential concerns. Upholding the ideal of maximum transparency at all stages is of prime importance.

In terms of GM, concerns are often raised about the stability of the GM constructs and the possibility of escape. Both are possibilities, however remote, whose risks need to be calculated and assessed. In principle, single‐ and tightly linked genetic units should be less resistant to recombination and hence breakdown than larger or multicomponent systems. It is also important to understand whether the ultimate consequences of such a breakdown are likely to be the inadvertent spread of introduced genes or gradual loss of the introduced genetic material. In general, risk mitigation and recall strategies for all GM methods are essential to consider from the initial proof of principle stage.

## A perspective from evolutionary biology

The general importance of bridging the gap between evolutionary biology and genetic pest management to develop effective and long‐lasting control strategies has been well recognized (Gould 2008). This dialogue can usefully inform the most effective way in which to target pests and to prevent the control strategies employed being degraded by the evolution of various forms of resistance.

The basics are straightforward and well understood; if we apply a selective pressure for any trait, then, given sufficient genetic variation, the population will respond. The response of the population to that selection pressure will be determined by the size of the selection differential (the difference in the mean value of the trait under selection in the original versus selected parent populations). The heritability of the trait under selection can be calculated by the ratio of the response over the selection differential (Falconer and Mackay 1996). The selection differentials that exist when wild strains are brought into the laboratory or factory can be huge – covering all aspects of life histories (Table**** 1). In effect, when pest strains are domesticated, a large‐scale artificial selection experiment is conducted upon the ability of individuals to survive and prosper in the novel environment. We should therefore expect released insects to have compromised performance when placed into field environments to which they are no longer adapted.

There are many ways in which the process of generating insects for control programs has the potential to result in selection for traits that are likely to lessen the effectiveness of released insects in the field. The life history consequences for laboratory selection in this context have been considered in some detail (e.g., Cayol 2000). What has been less well implemented are strategies to tackle them, even though with adjustments to husbandry practices such effects could, in principle, be circumvented or minimized (Table**** 1).

The general consequences of domestication are a significant reduction in genetic diversity. The initial stages of domestication often involve a fairly savage bottleneck, which can significantly reduce genetic diversity in comparison with wild progenitor populations. This increases the net effect of genetic drift and the likelihood that rare gene variants important for success in the field may be lost. However, unless the bottleneck is particularly drastic, of greater importance are changes in allele frequencies that subsequently occur due to strong selection for domestication. A proposed solution to this problem is to conduct periodic introduction of wild individuals into the domesticated strains (the ‘fresh blood’ technique). This would help to reduce the impact of many of the concerns listed in Table**** 1, although increases in the genetic diversity, and any associated benefits, may be temporary as wild alleles are likely to be selected against in the laboratory or factory environment. An associated problem is the loss of genetic diversity in the accompanying microbiome of the domesticated individuals. There are obvious cost implications of the potential solutions above as they decrease productivity. However, it is also important to consider that little work has yet been performed on the relationship between the improvements suggested above and the gain in control effectiveness.

Comprehensive stress testing of GM strains is crucial to prevent unwanted surprises down the line. For example, the effect on strain stability of temperature, food availability, humidity, and pathogens should all be examined. For control mechanisms in which the sex‐specific lethality relies upon the absence of dietary additives, it must be clear that such additives cannot be encountered at anything close to an effective dose by the released insects in the field or urban settings. Further insight into predicting the likely effectiveness of released insects may also come from a better integration of population genetics. For example, understanding population genetics, gene flow, and the effects of partial reproductive isolation are important for understanding the impact and efficiency of release programs (e.g., Endersby et al. 2011).

### Understanding sexual selection and the mating biology of pests is crucial to improving control via GM and non‐GM technologies

As emphasized above, the production of safe and fit insects for release is key to success of all SIT and GM technologies (Scolari et al. 2011). One important lesson relevant to all control strategies discussed above, both GM and non‐GM, is that knowledge of the life history and reproductive biology of the pests involved is as important now as it has ever been (e.g., Briceño and Eberhard 1998; Briceño et al. 2002; Leftwich et al. 2012; Oliva et al. 2013; Perez‐Staples et al. 2013). Important insights into the success of SIT and genetic control programs have come from knowledge of compounds that affect male mating behavior (e.g., Kouloussis et al. 2013), the best attractants for traps, and the effect of sterilization on mating and remating behavior (Kraaijeveld and Chapman 2004). Direct comparisons between the fitness and competitiveness of strains carrying GM technologies versus controls and the wild‐type populations remain an essential part of the toolkit for validation of these technologies (e.g., Morrison et al. 2009; Massonnet‐Bruneel et al. 2013; Leftwich et al. 2014). Also of great importance is knowledge of the effects of domestication on the control potential of released insects. This knowledge can be used to minimize the effects of selection for traits that compromise control efficiency.

Trapping and detection methods are also key to successful insect control and just as important to the successful implementation of genetic control methods as the basic genetic technology itself. Therefore, continuing research into attractants is important to gain knowledge into the incidence and distribution of pest populations, and to easily and reliably detect released versus wild insects in the field (e.g., Juan‐Blasco et al. 2013). This includes theoretical and empirical investigations of the effects of environmental factors (Dufourd and Dumont 2013). Understanding of the dispersal of released insects is also important to predict the likely effectiveness of insect control from released programs (e.g., Gavriel et al. 2012). To date, there has been little consideration of the age structure of the population into which insects will be released. This is of particular importance if there is any assortative mating by age and can affect the numbers of released insects likely to give effective control (Huang et al. 2009). Future empirical research into these factors may be useful.

We conclude that ultimately it should be possible to minimize the impact of natural selection on the effectiveness of insect control by SIT and GM methods by understanding what are the principal and key elements of reproductive success of the pests in the natural environment and building the understanding of that knowledge into rearing practices.

## Control strategies made evolution‐proof or evolution‐resistant

Evolution is inevitable given the existence of genetic variation, and, given this, evolution by natural selection is also assured whenever a selective force is applied. The evolution of resistance to control strategies of all kinds is, therefore, inevitable. Control programs cannot be made evolution‐proof, but the deleterious impact of natural selection on control efficiency can be substantially mitigated. There is recognition that combinations of simultaneous and diverse approaches are needed to prevent degradation in the effectiveness of individual approaches over time (Deredec et al. 2008; Alphey 2014). However, there has been very little exploration to date of the most efficient combinations of genetic techniques for insect control. A combination of approaches is needed not just to spread risk in a general sense, but to diffuse the strength of natural selection focused on specific traits likely to diminish the effectiveness of control.

We suggest that there is much to learn from the study of insecticide, chemotherapy, and antimicrobial resistance (AMR). AMR in particular is a grand challenge, representing a major global threat to human health in terms of our ability to combat infectious disease as well as to treat cancer via chemotherapy. Although the contexts are different, the underlying principles of how to slow the spread of resistance are conceptually similar as they all rely upon the same evolutionary principles. It has also been recognized that facets of resistance are predictable according to mechanisms of resistance and the environment in which resistance evolves. Therefore, an approach that integrates across these levels is needed (Maclean et al. 2010). Thinking across insecticide resistance and AMR has led to the proposal of four major strategies to slow and manage the evolution of resistance (REX consortium, 2013) as outlined below.

1. *Responsive alternation* refers to the strategy of sequential use, applying different control methods in series (but not cycling them). For example, one method might be applied continually until resistance is observed and then the next method applied.
2. *Periodic application* is when control methods are cycled or rotated; hence, a pesticide might be used for 6 weeks then a second used then back to the first. Note that in methods (1) and (2), the application of control varies over time but not space (i.e., is uniformly applied everywhere).
3. *Mosaic* is an approach that varies space but not time. For example, at least two different control methods are applied simultaneously but in different places and the places in which they are used do not overlap. An example might be the use of different antibiotics in different hospitals or different pesticides in different fields.
4. *Combination* is when 2 or more approaches are applied concomitantly over time and space. An example is the use of combinatorial therapy for HIV infection, with multiple drugs being applied simultaneously.

Variation of all of these approaches using full‐ and half‐strength control strategies is also possible. Allied to the thinking that less than total eradication might be useful is recent research into the need to prevent chemotherapy resistance, which suggests that managing cancer, rather than eradicating it, may sometimes be a more successful strategy overall (Greaves 2007; Read et al. 2011).

A recent review of the efficacy of these methods applied across very different contexts in medical and agricultural settings (REX Consortium 2013) suggests that, in terms of their ability to slow the evolution of resistance, *combination* methods were best, outperforming *periodic application* and *mosaic* approaches (which were equivalent) and all were better than *responsive alternation*. The *combination* approach works well because it ensures that individuals are killed even if they are resistant to one of the approaches applied.

The basic underlying principle is to create scenarios in the target population (be it microbes, insects, or crops) in which there is greater variation in selection pressure on the pest to evolve resistance. This strategy will ultimately give rise to more sustainable pest control over the long term. Imposing variation in selection pressure for resistance is important because it presents a less strong but, more importantly, a moving target. Encouragingly, initial suggestions for combinatorial approaches are being made. For example, Deredec et al. (2008) suggest that the evolution of resistance to HEGs could be slowed by simultaneously targeting multiple genes using multiple HEGs, or by targeting multiple sites within the same gene. HEG constructs should also be rigorously designed to reduce the probability that the expression of the gene product becomes separated from the recognition site. Other possibilities are to combine RIDL systems that employ female‐specific lethality with releases of engineered males susceptible to other control methods (e.g., to insecticides or to *Bacillus thuringiensis* (*Bt*)‐engineered toxins expressed by GM crops). Such methods could provide dilution of resistance across the different mechanisms (Alphey et al. 2007; Alphey et al. 2009; Alphey et al. 2011). Genes introduced into wild populations by released males will be inherited by males in systems that employ female‐specific lethality and by both sexes if resistance permits some progeny to survive the effects of the engineered ‘lethal’ genes. Theory suggests that inheritance of susceptibility genes through this mechanism can slow or potentially reverse the spread of resistance mutations to RIDL, prolonging the effectiveness of this technology (Alphey et al. 2011). This resistance dilution would potentially work for release programs, such as RIDL or SIT, in which releases are sustained over time, but is not expected to occur in drive‐based systems that employ limited, inoculative releases.

An important consideration for combination approaches, should they be adopted for insect control, is that SIT and GM approaches have well‐documented fitness costs, as outlined above (e.g., costs of bearing GM constructs, loss of fitness upon irradiation, costs of bearing *Wolbachia* infection). Such fitness costs incurred simultaneously under a combination approach have the potential to impose a greater fitness ‘load’ upon the release population and potentially reduce its effectiveness. These costs would therefore have to be weighed up against the advantages. Fitness costs to released insects of SIT and GM technologies have been mentioned in several different contexts, and their magnitude is a key determinant for successful control. Under a traditional model in which there is a fixed resource pool that can be allocated to different life history traits but which cannot maximize them all simultaneously. The costs of bearing a GM construct or driving strain of intracellular microorganism are therefore likely to lead to trade‐off with other life history traits with effects on fitness.

The need to recognize and minimize resistance has not yet permeated deeply into discussions of SIT and GM insect control. An approach similar to responsive alternation is sometimes used in SIT programs – for example, pesticides may be used to reduce initial population sizes before SIT intervention. Combination control has, though, been used in other agricultural contexts. GM crops engineered using *Bt* technology have been developed that produce several different toxins against their target pests (Cui et al. 2011). A *combination approach* involving the use of *Bt* crops and sterile insect releases to target pink bollworm (*Pectinophora gossypiella*) removed the need for insecticide sprays and was effective at reducing pest abundance while maintaining current resistance levels to *Bt* cotton (Tabashnik et al. 2010).

### Improved targeting of insect control

Consideration of the problems created by the blanket use of broad spectrum antibiotics that has hastened in the current potentially catastrophic problem of AMR has led to increased interest in improved diagnostics coupled with the use of newer narrow spectrum (highly selective) antibiotics. Such a strategy facilitates the use of combination therapies discussed above.

Translating this into the control strategies considered here, diagnostics would equate to developing a better understanding of the pest problem (its ecology, population dynamics, fluctuation, location, intensity), and narrow spectrum antibiotics to an understanding (based upon the diagnostics) of which selection of diverse GM and non‐GM specific control strategies available could be targeted most effectively. There is no reason why this new thinking on rapid, point‐of‐need strategies combined with better stewardship could not, in principle, be applied in terms of GM technologies for control. We offer some general thoughts on principles to maintain fitness and competitiveness of control strains and hence increase effectiveness of control programs (Box**** 1).

Box 1 **General principles for maintaining fitness and competitiveness of control strains and increasing effectiveness in control programs**

- Keeping the domesticated progenitor and GM strains in an outbred genetic background with frequent outcrossing to promote the maintenance of a wild‐type ancillary genome.
- Keeping the domesticated environment as complex and varied as is feasible.
- Diet variation and supplementation may be useful to maintain variation in traits related to nutrient acquisition and to maintain diverse gut microbiomes.
- Knowledge of the ecology, life history, and reproductive success of wild‐type strains is essential to inform best practice in husbandry and in trapping technology.
- Simple GM constructs and vehicles seem more likely to be stable and hence less likely to break down than more complex ones.
- Drive systems should have built in safeguards.
- Theory, parameterized by real world data, is essential to predict and test program‐specific optimal invasion thresholds, release ratios, release frequencies, release timing (with respect to season and resident population size), release population composition (e.g., age structure).
- Strategies from the study of insecticide resistance and antimicrobial resistance (AMR) could lead to improved strategic and combined deployment of GM and non‐GM strategies.

## Insect control and conservation

In this final section, we conclude by discussing briefly an emerging idea that GM technologies for insect control are not necessarily in conflict with modern conservation planning. These research areas have typically proceeded along very separate lines, but dialogues led by new thinking in conservation practice may offer opportunities for synergy. For example, recent research in conservation has advanced the controversial idea that ‘land sparing’ has the potential for greater conservation value than does ‘land sharing’ (Phalan et al. 2011, 2014). Under this scenario, there is greater preservation of biodiversity through the intensification of farming on existing land. This is because it allows for less land to be used for the same yield and therefore more land to be freed up to return to its natural state, or be preserved, and support a greater number and diversity of natural species than is true under other conservation scenarios. Increases in productivity in the order of a few % per annum could support this scenario and are predicted to be possible. Control of agricultural pests using GM technologies could play a role under this scenario. They allow relatively cost‐effective and targeted control of insect pests with less environmental impact than is true for pesticides. This sets up the interesting situation that rather than being in opposition to the preservation of biodiversity, the development of advanced GM technology could actually be part of the solution to preserve it. Future work on integrating the likely efficiency savings for yield of the application of GM control programs would be especially useful to ground truth these interesting ideas.

## Author contributions

TC wrote the first draft of the manuscript, and all authors contributed to editing and revisions.

## Data archiving statement

There are no data to be archived.
